# Chemical RNA Cross-Linking: Mechanisms, Computational Analysis, and Biological Applications

**DOI:** 10.1021/jacsau.2c00625

**Published:** 2023-01-16

**Authors:** Willem A. Velema, Zhipeng Lu

**Affiliations:** † Institute for Molecules and Materials, Radboud University, Nijmegen 6500 HC, The Netherlands; ‡ Department of Pharmacology and Pharmaceutical Sciences, School of Pharmacy, University of Southern California, Los Angeles, California 90033, United States

**Keywords:** RNA, Oligonucleotides, Cross-linking, Nucleic-acid Chemistry, RNA-seq, RNA Structure

## Abstract

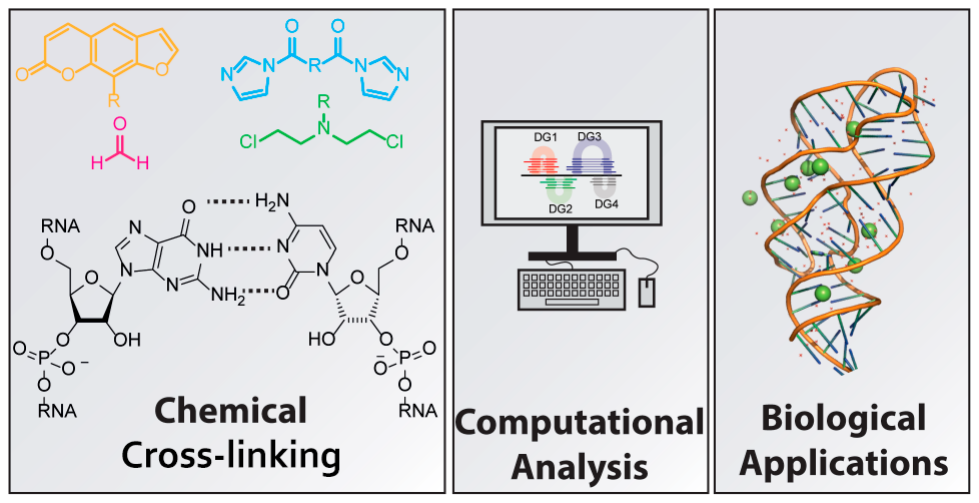

In recent years, RNA has emerged as a multifaceted biomolecule that is involved in virtually every function of the cell and is critical for human health. This has led to a substantial increase in research efforts to uncover the many chemical and biological aspects of RNA and target RNA for therapeutic purposes. In particular, analysis of RNA structures and interactions in cells has been critical for understanding their diverse functions and druggability. In the last 5 years, several chemical methods have been developed to achieve this goal, using chemical cross-linking combined with high-throughput sequencing and computational analysis. Applications of these methods resulted in important new insights into RNA functions in a variety of biological contexts. Given the rapid development of new chemical technologies, a thorough perspective on the past and future of this field is provided. In particular, the various RNA cross-linkers and their mechanisms, the computational analysis and challenges, and illustrative examples from recent literature are discussed.

## 1 Introduction

RNA is increasingly being recognized as a versatile biomacromolecule involved in virtually every process in the cell.^1,2^ In addition to serving as a passive carrier of genetic information like DNA, RNA can fold into complex structures like proteins.^3^ The various structures formed by RNA, either within a single RNA molecule, or between different RNAs, carry out diverse and active cellular functions, such as catalysis, scaffolding, and guiding.^1,4^ Normal and abnormal functions of RNA underlie a variety of human pathologies, such as virus infections, nucleotide repeat disorders and splicing diseases.^5^ Technological advancements in several fields have culminated in new RNA-based and RNA-targeting therapeutic approaches in recent years, including small molecule drugs that target highly structured regions,^6−8^ antisense oligos that target low structured regions or alter RNA structures,^9^ and mRNA therapies based on modification chemistries that stabilize RNA and minimize innate immunogenicity.^10^

**Figure 1:**
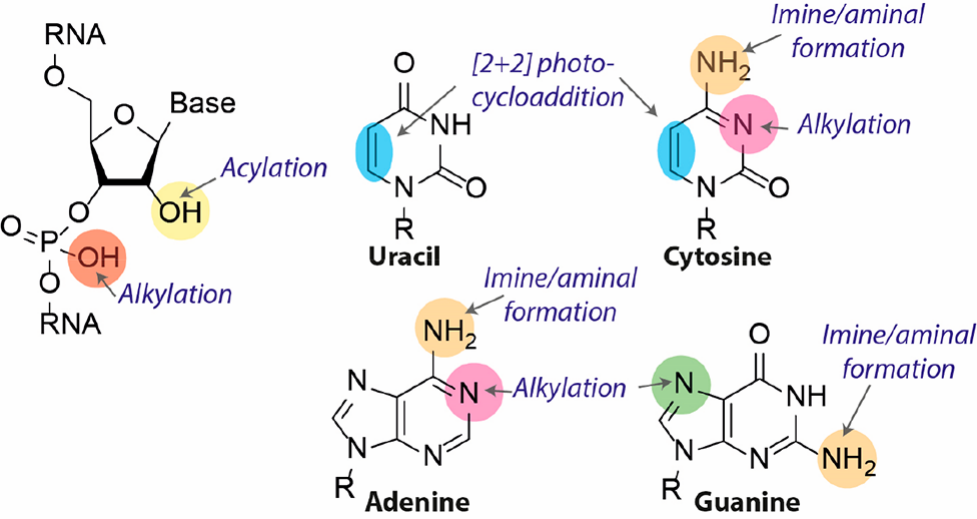
Molecular structure and reactive sites of RNA. The phosphate group (red) can be alkylated with diazo compounds. The 2′-OH (yellow) is prone to acylation. The C5–C6 double bond in uracil and cytosine (blue) can undergo a photocycloaddition. The exocyclic amines (orange) can react with aldehydes to form an imine or aminal. The N7 position of guanine (green) can be alkylated as well as the N1 of adenine and N3 of cytosine (pink).

To dissect the critical roles of RNA in biology, to study disease mechanisms, and to develop RNA-targeting therapeutics, structural analysis of RNA has played an essential role.^11^ While conventional physical methods, such as NMR, X-ray crystallography and cryo-EM have been instrumental in protein structure analysis, their applications in RNA have been more limited, primarily due to RNA’s large size, high flexibility, and strong dependence on physiological environments.^11,12^ Computational structure modeling based on minimal free energy calculations and phylogenetic analysis of conservation and covariation also suffer from multiple limitations, such as lack of understanding of RNA folding rules and high computational cost. Therefore, direct measurements of RNA structures in cells have been critical for understanding RNA behavior in various biological and pathological processes.^13^ A variety of chemical reactions have been developed and exploited that can modify RNA at certain positions (Figure 1) depending on nucleotide reactivity, flexibility, or accessibility, which correlate with RNA structural constraints.^14^ For example, dimethyl sulfate (DMS) selectively alkylates the N1 position of adenine and N3 position of cytosine on unpaired nucleotides^15^ and the 2′-OH in flexible regions can be acylated with Selective 2′-Hydroxyl Acylation analyzed by Primer Extension (SHAPE) reagents.^16−18^ Resulting reactivity profiles have been useful to improve secondary and tertiary structure modeling;^19−21^ however, the 1D information obtained with these experiments is not necessarily definitive evidence for specific structures. More recently, correlated chemical probing coupled with computational deconvolution has been used to discover potential contacts and alternative conformations in relatively short RNA regions and simple conformations.^22−24^

In theory, the structure of any object can be uniquely determined by the coordinates of their components, which should be equivalent to the distances among the components. Therefore, measuring the spatial distances between nucleotides should allow de novo determination of RNA structures in any biological sample. Chemical cross-linking of RNA in cells using compounds of defined sizes and subsequent identification of cross-linked nucleotides represents a practical path toward this goal. Pioneering work by Hearst and colleagues in the 1970s and 1980s^25−28^ demonstrated this principle using psoralen, a duplex-specific agent that cross-links opposing pyrimidine nucleobases with two consecutive [2 + 2] photocycloadditions.^29^ Subsequent applications provided the first physical evidence for snRNA-dependent pre-mRNA splicing, and snoRNA-guided rRNA processing.^12,30−32^ Rapid progress in both cross-linking chemistry and sequencing technology in the past few years have led to several new high-throughput methods for the analysis of RNA structures and interactions.

While other recent reviews have summarized these methods,^12,14,33−35^ a critical analysis and evaluation of the chemical mechanisms, computational tools, and applications in biology have been lacking. Simultaneous consideration of these three aspects is necessary to solve increasingly challenging problems in RNA chemistry and biology. In this perspective, we focus on these topics together, to inspire chemists to further develop specialized chemical cross-linking agents necessary to tackle outstanding questions in RNA biology and provide a guide for biologists to choose the most appropriate methods for addressing specific biological questions.

## 2 Chemistry of Current Technologies

### 2.1 RNA Chemical Reactivity

The molecular structure of RNA contains multiple functional groups that can potentially be targeted with (photo)chemical reagents to form cross-links (Figure 1) of which the more common ones are described here. The phosphodiester in the RNA backbone can be alkylated with diazo compounds (red, Figure 1),^36^ which has been successfully demonstrated with caging agents to control RNA function.^37,38^ Gillingham and co-workers showed that terminal phosphates are more prone to O-alkylation with diazo compounds than internal phosphate diesters, for which usually a large excess of reagent is required. This was attributed to the substantial difference in p*K*_a_ (∼6–7 for phosphate monoester and ∼1 for phosphate diester).^36^ The 2′-OH position (yellow, Figure 1) can be acylated with activated carbonyl reagents, which forms the basis of SHAPE, a widely used method to determine RNA secondary structure.^16,17,39^ More recent bifunctional acylators target the 2′-OH position to establish cross-linking.^40−42^ The unusual nucleophilicity of the 2′-OH position has been attributed to oxyanion formation at this position due to inductive effects provided by neighboring 3′ and 4′ oxygens and the nucleobase nitrogen.^43,44^ Pyrimidine bases can undergo [2 + 2] photocycloadditions using their C5–C6 double bond (blue, Figure 1).^28^ This feature has been extensively exploited with psoralen cross-linkers.^29^ More recent photochemical cross-linkers such as carbazoles and coumarins undergo photocycloadditions as well with improved efficiencies.^45^ The exocyclic amines of cytosine, adenine and guanine (orange, Figure 1) can react with aldehydes to form imines.^46^ When using formaldehyde an aminal bond is formed that cross-links opposing nucleobases.^46^ The N7 position of guanine (green, Figure 1) can act as a nucleophile toward nitrogen mustards.^47^ Interestingly, the N7 position of adenine is unreactive toward nitrogen mustards, which is mainly attributed to the lower nucleophilicity as compared to the N7 of guanine.^47,48^ The N1 of adenine and N3 of cytosine (pink, Figure 1) can act as nucleophiles toward appropriately electrophilic compounds.^15^ This is mainly exploited with DMS footprinting^15,49,50^ to elucidate RNA secondary structures. Moreover, the N3 position of guanine and uracil can also be methylated by DMS, but at lower rates causing this reactivity to be mainly ignored.^51^

We make a distinction between external and internal cross-linking reagents. External cross-linking reagents are the main focus of this perspective and can be added exogenously to samples without the need of first modifying the RNA under investigation, which allows the study of native RNA. Other advantages are that they are generally more accessible and pan acting, thus enabling transcriptome-wide interrogations. Conversely, internal cross-linking reagents are installed in the RNA scaffold prior to the study either chemically or enzymatically and include coumarins,^52^ carbazoles,^45^ thionucleotides,^53^ diazirines,^54^ and platinum complexes^55^ among others and have recently been reviewed.^56^

**Figure 2:**
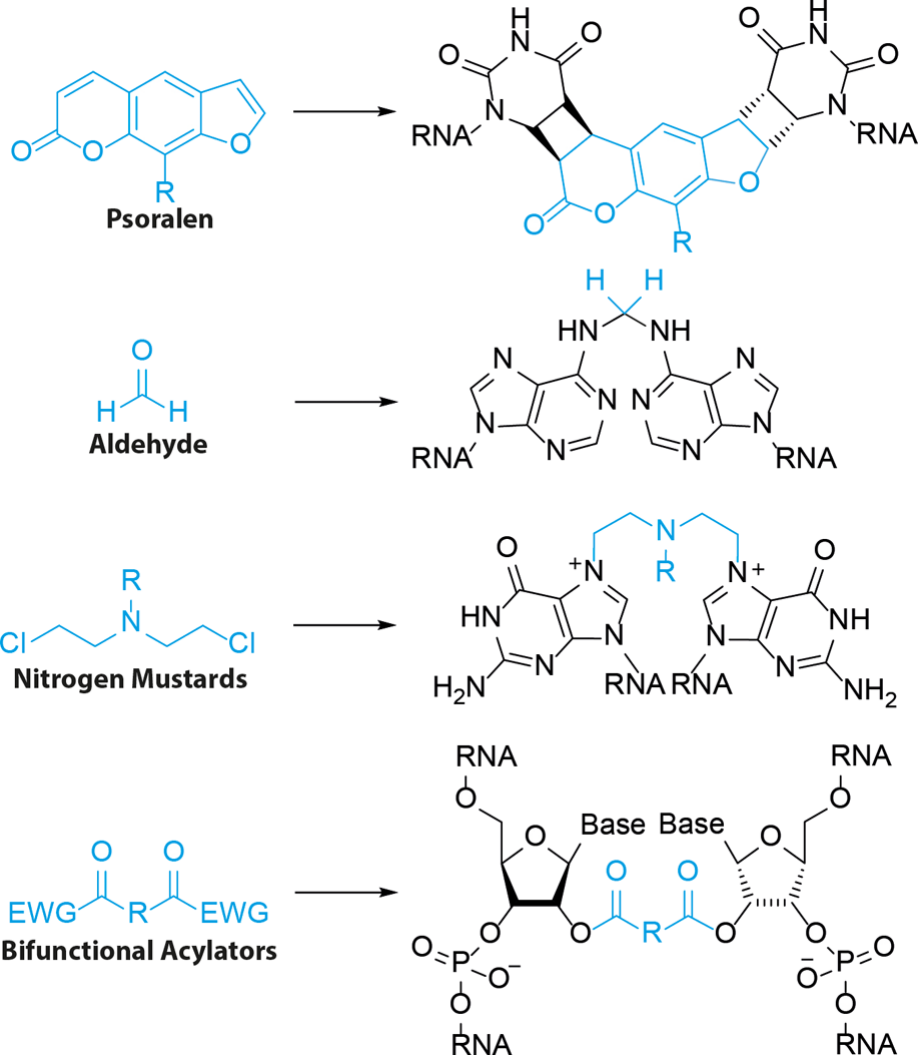
Molecular structure and corresponding RNA adducts of the most commonly employed external RNA cross-linking reagents.

Four classes of external cross-linking reagents (Figure 2) are predominantly used, and each offers unique advantages and reacts with distinct functional groups on the RNA scaffold to establish intra- or intermolecular cross-links.

### 2.2 Psoralen Cross-Linkers

**Figure 3:**
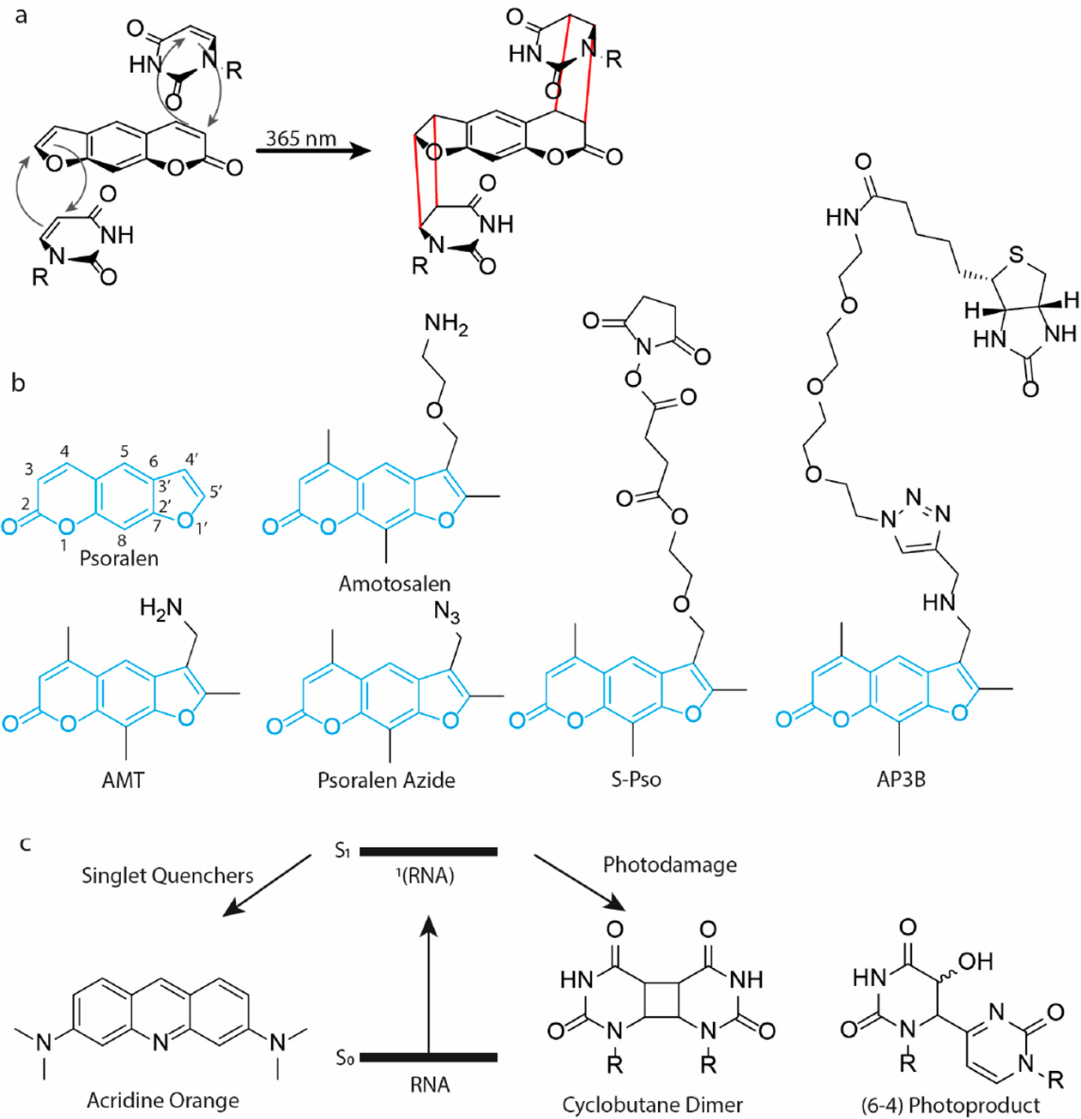
Psoralen cross-linkers. (a) Cross-linking mechanism. Psoralen intercalates between opposing pyrimidines. UV exposure initiates a [2 + 2] photocycloaddition between the C5–C6 double bond of uracil or cytosine and the 3,4 and 4′,5′ double bond of psoralen to form a cyclobutane and cross-linking the pyrimidines. (b) Molecular structure of psoralen derivatives. (c) Singlet quenchers like acridine orange can protect RNA from photodamage.

One of the most commonly employed RNA cross-linkers for structural studies is psoralen.^25^ This natural product can undergo two consecutive [2 + 2] photocycloadditions to cross-link opposing pyrimidine bases (Figure 3a).^28^ and several modified versions have been reported to study RNA–RNA interactions (Figure 3b). Psoralens first intercalate into duplex regions, placing the reactive 3,4 and 4′,5′ double bonds in a favorable position toward the C5–C6 double bond of pyrimidine bases. Exposure to 365 nm light results in a photocycloaddition.^28^ Both the 3,4 and 4′,5′ double bonds can in principle react first, however only 4′,5′ adducts still absorb at 365 nm and can therefore undergo a second photocycloaddition with the 3,4 double bond resulting in a successful cross-link.^25,57^ Importantly, this cross-link can be reversed by exposure to ∼250 nm light, which is heavily exploited in RNA structural studies to simplify data analysis. This reversal reaction has reported quantum yields between 0.16 and 0.30, depending on the wavelength.^26^ The furan-side was more readily reversed than the pyrone-side with an ∼2-fold difference in rate at pH 2.2 and ∼20-fold difference at pH 7.5.^26^ In practice, the reversal reaction has been reported with limited efficiency,^40^ which in part was attributed to significant RNA degradation.^58^ A base-catalyzed rearrangement has been reported with higher efficiency that selectively cleaves the cross-link at the pyrone side of psoralen.^59^ To overcome RNA damage including potential cyclobutane dimers and (6-4) products (Figure 3c) due to UV exposure caused by psoralen (un)cross-linking, Lu and colleagues developed a protocol^60^ based on acridine orange (Figure 3c) singlet-state quenching.^61^ They demonstrated that 30% of RNA remains intact after 30 min irradiation with 254 nm at 4 mW/cm^2^ in the presence of acridine orange, compared to less than 0.5% in its absence, enabling efficient application of psoralen cross-linking for RNA structure determination.

Over 100 psoralen derivatives have been reported with improved photophysical, physicochemical and biochemical properties as compared to the parent compound.^25^ For example, AMT (Figure 3b) bears a primary amine that improves solubility in water at physiological conditions to ∼1 mg/mL, enabling application for RNA structural studies, first reported by Calvet and Pederson to study heterogeneous nuclear RNA (hnRNA) in live cells.^62^ Using AMT they found that hnRNA contains double stranded regions that are organized in an accessible manner within the nucleus. AMT efficiently cross-linked double stranded regions in cells with a 7.2-fold increase as compared to UV light alone.^62^ With recent revolutionizing developments in Next Generation Sequencing and bioinformatic analysis the use of AMT to determine RNA structure on a transcriptome-wide level has increased. Psoralen Analysis of RNA Interactions and Structures (PARIS) and LIGation of interacting RNA followed by high-throughput sequencing (LIGR-seq) used AMT cross-linking to map transcriptome-wide RNA structures and discover previously unknown RNA–RNA interactions.^63,64^

To further improve the analysis of dynamic RNA structures and interactions, PARIS2 uses amotosalen (Figure 3b) instead of AMT for cross-linking.^60^ The limited solubility of AMT at ∼1 mg/mL in water was hampering the efficiency in capturing RNA–RNA interactions. Amotosalen bears a primary amine and ether group and exhibits markedly increased solubility in water of 230 mg/mL. In vivo, it was found that a 10-fold increase in concentration (0.5 mg/mL AMT vs 5.0 mg/mL amotosalen) resulted in a 7-fold increase in cross-linked RNA. This overall improved cross-linking allowed for establishing the first whole genome structure of enterovirus D68 and dynamic interaction networks for the U8 snoRNA, where genetic mutations cause a neurological disorder.

To increase the functionality of psoralens, azido modified derivatives were designed that can undergo azide–alkyne cycloadditions. For example, Hall and co-workers used Psoralen Azide (Figure 3b) to append to the 3′-end of alkyne modified pre-microRNAs with up to 94% conjugation yield.^65^ A different azido derivative bears the azide group at the end of a triethylene glycol linker attached to the primary amine of AMT and forms the basis of Cross-linking Of Matched RNA And Deep Sequencing (COMRADES).^66^ Using this psoralen derivative, cross-linked RNA can be selectively captured and enriched using a biotin ligation and streptavidin pulldown.^67−69^ The presence of the azide group did not affect the cross-link efficiency and using this method the researchers determined the architecture of Zika virus inside cells.^66^

To conjugate psoralen to an RNA of interest, Rana and co-workers designed the NHS-ester bearing derivative S-Pso (Figure 3b).^70^ This was ligated to an amine bearing miRNA-29a mimic using standard amide-coupling conditions to afford conjugated RNA in quantitative yields. After transfection into HeLa cells, S-Pso labeled miRNA-29a efficiently silenced luciferase gene expression of a reporter plasmid containing a miRNA-29a target site in its 3′ UTR to ∼15%, showing that the psoralen group did not affect miRNA silencing. The miRNA mimic was then applied to identify targets in live cells. After photo-cross-linking, captured transcripts were quantified by RT-qPCR. Tet2 RNA was identified as a miRNA-29a target with 20-fold enrichment after cross-linking. This study highlights the potential use of psoralen cross-linkers in RNA target identification.

Biotinylated psoralen would allow for direct enrichment of cross-linked RNA using streptavidin beads. Taking advantage of this, Nagarajan, Wan, and co-workers^71^ developed Sequencing of Psoralen cross-linked, ligated and Selected Hybrids (SPLASH). In particular the sensitivity of their method increased from ∼0.45 with psoralen to ∼0.75 with biotinylated psoralen. Using SPLASH the authors identified hundreds of known and unknown snoRNA-rRNA binding sites. One disadvantage of this protocol is that cellular uptake of biotinylated psoralen was low, and 0.01% digitonin was added to increase uptake. The psoralen derivative used in this study was modified at the C8 position with biotin. Lin and co-workers found that when biotin is appended to the 4′-position of AMT (AP3B, Figure 3b), the efficiency and cross-linking is significantly improved.^72^ Using a gel-shift assay and dsDNA in vitro, a 100-fold increase in efficiency was observed. In cells a 5-fold increase in biotinylation of DNA was observed. The cross-linking efficiency for RNA was not determined.

### 2.3 Aldehyde Cross-Linkers

**Figure 4:**
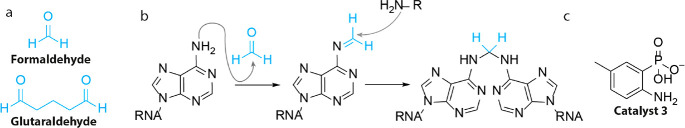
Aldehyde cross-linking. (a) Structure of formaldehyde and glutaraldehyde. (b) Cross-linking mechanism of formaldehyde. (c) Molecular structure of **catalyst 3** that reverses formaldehyde cross-links.

The use of aldehydes to cross-link biological samples dates back to the 19th century, when Blum reported successful tissue fixation with formaldehyde (Figure 4a).^73^ Formalin-Fixed Paraffin Embedded (FFPE) treatment is used for preservation for long-term storage and preservation of patient samples. Glutaraldehyde (Figure 4a) has been used extensively for cross-linking proteins,^74^ but studies with RNA are scarce, although it is likely that it can cross-link nucleic acids. The dialdehyde glyoxal has recently been used as well for temporary caging of nucleobases^75^ and when incorporated in the backbone of DNA it was shown to cross-link to proteins.^76^ Formaldehyde forms adducts and cross-links with biomolecules that protects them from degradation. Specifically, formaldehyde can react with the exocyclic amines of adenine, guanine and cytosine to form imine and hemiaminal adducts and aminal cross-links (Figure 4b).^46,77,78^ The first step is rapid, but the formed imine inhibits base pairing interactions, significantly slowing down cross-linking, which requires the second nucleophile to be in close proximity.^79^

The formed adducts are in principle reversible and allows for analysis of genetic material after long-term storage.^80^ After RNA extraction, samples are typically incubated at 80–90 °C in Tris buffer for several hours, which reverses hemiaminal formation. These relatively harsh conditions have been shown to affect the integrity of RNA, impeding meaningful quantification of RNA levels. Kool and co-workers designed catalysts that can speed up adduct removal, yielding higher quality RNA. In particular phosphanilate **catalyst 3** (Figure 4c) efficiently reversed hemiaminal adducts. Incubation at 5 mM for 2 h reversed ∼50% of adducts compared to 11% without catalyst. While recovering adducts from FFPE prepared cell samples, up to 25-fold enhancement in recovery was found for **catalyst 3** compared to no catalyst, as was quantified with qRT-PCR. Longer RNA amplicons benefited most from catalytic adduct removal. It was suggested that the mechanism of catalyst-assisted reversal is based on general acid catalysis with possible nucleophilic catalysis.

Apart from RNA preservation, aldehyde cross-linking can be used for mapping RNA–RNA interactions as well. Guttman, Lander, and co-workers exploited this to study U1 small nuclear RNA and the large ncRNA Malat1.^81^ The authors combined AMT (Figure 3b) cross-linking with formaldehyde cross-linking and found that both methods yield different RNA–RNA interactions: AMT provided information on duplexed interactions at high resolution, while formaldehyde could capture a broader range of interactions. After cross-linking, RNAs of interest were captured with antisense oligonucleotides and analyzed with high-throughput sequencing. Strong enrichment of target RNAs (>1000-fold) was obtained with both AMT and formaldehyde cross-linking. Using this approach, it was found that U1 RNA targets 5′ splice sites throughout introns and Malat1 interacts with pre-mRNAs mostly through protein intermediates.

### 2.4 Nitrogen Mustards

**Figure 5:**
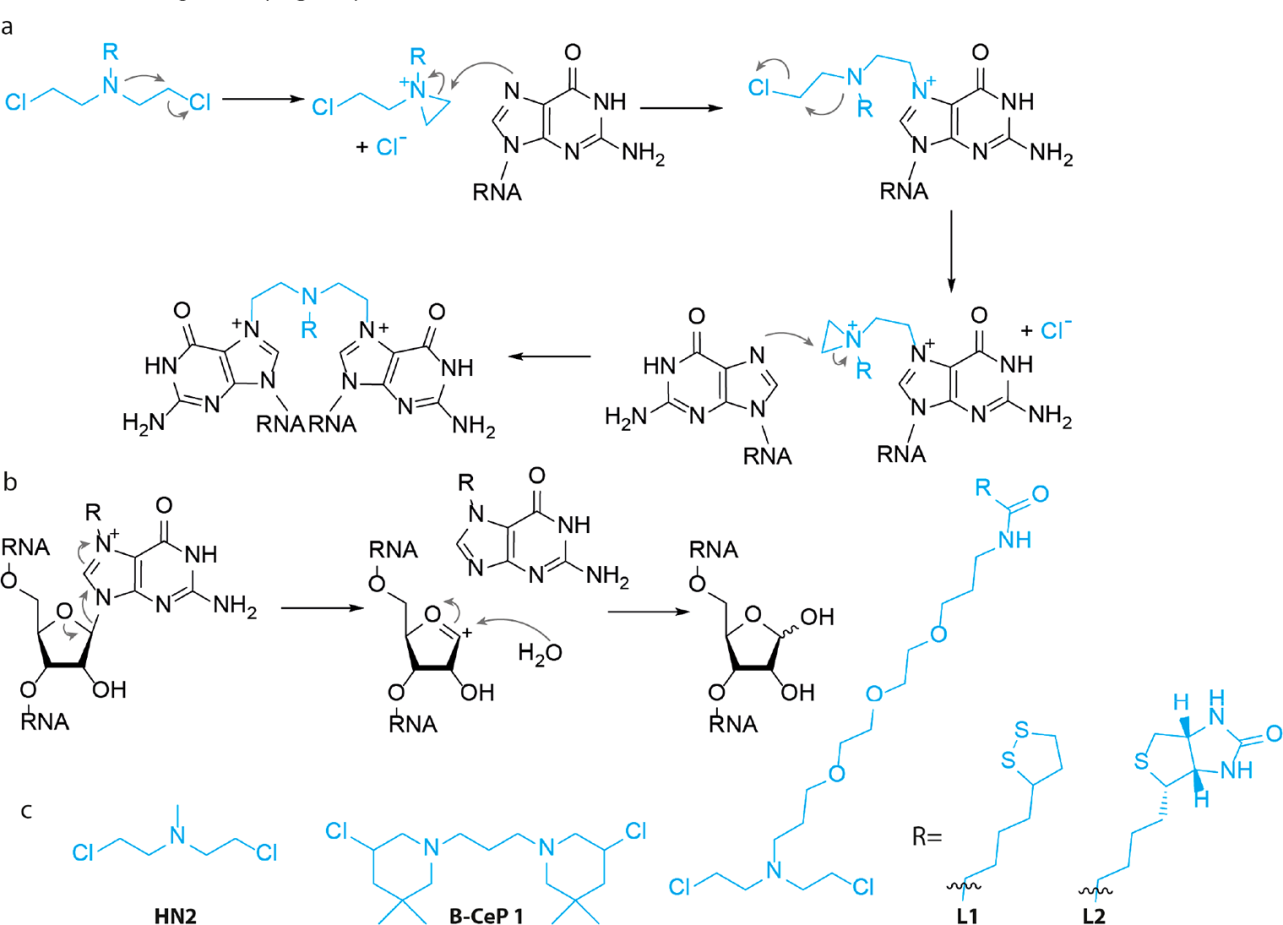
Nitrogen mustards. (a) Cross-linking mechanism. An aziridinium is formed that is attacked by the N7 nitrogen of guanine. Two consecutive reactions result in intra- or intermolecular cross-links. (b) Depurination mechanism. N7-alkylated guanine is electron poor, promoting cleavage of the N-glycosidic bond. (c) Molecular structure of nitrogen mustard derivatives.

Originally studied in the 1940s for their anticancer properties, nitrogen mustards have been found to be potent DNA cross-linkers and as such have found wide clinical use. This extraordinary feature was quickly realized to be applicable to cross-link RNA to investigate RNA–RNA interactions^82,83^ and study small molecule-RNA interactions.^84,85^ Nitrogen mustards are first activated by forming an aziridinium cation and elimination of chloride (Figure 5a).^47^ The aziridinium cation is sequentially attacked by the N7 of guanine resulting in alkylation. Two subsequent attacks lead to a bifunctional adduct, cross-linking RNA (Figure 5a).

One potential consequence of N7 alkylation is depurination. Many studies have investigated this mechanism in DNA,^86^ which involves breakage of the N-glycosidic bond promoted by the positive charge and subsequent reaction with water (Figure 5b).^87^ N7 alkylated guanine was shown to depurinate 10^6^ more rapidly than guanine under physiological conditions.^88^ RNA is considered less prone to depurination, because the 2′-OH destabilizes the oxocarbonium intermediate,^87^ but several cases in RNA have been reported,^89^ which could hamper interpretation of obtained data. There appears to be a physiological relevance to this reaction and enzymatic repair pathways have been identified.^90^

In one of the first reports, the authors applied **HN2** (Figure 5c) to study ribosomal subunits in *Escherichia coli* (*E. coli*). It was first attempted to cross-link 16S and 23S RNA using UV cross-linking, but no interactions were found. **HN2** did efficiently cross-link 16S-23S RNA as analyzed by gel electrophoresis, which led to the conclusion that there are several RNA–RNA interactions within the ribosomal particle and that these can be explored with chemical cross-linking methods. Datta and Weiner applied this principle to investigate higher order RNA structures and tertiary interactions.^91^ Nuclear extracts were subjected to 20 mM solutions of **HN2** and analyzed using sequencing gels. Intramolecular cross-links in U2 snRNA were apparent and could be localized within regions of a few nucleotides. Exact pinpointing of cross-link sites was hampered by monoadducts. Nevertheless, the data clearly supported a tertiary structure model for U2 snRNA.

Using mass spectrometry (MS), Fabris and co-workers analyzed **HN2** cross-linking sites in HIV-1 SL1A RNA.^92^ This structured RNA contains a large flexible loop and an internal bulge. Cross-linking was performed with 250 μM HN2 and subsequently digested with RNase A. Using MS, cross-linked fragments were observed that originate from both the loop and bulge region. Interestingly, guanine-adenine cross-links were observed as well, implying that nitrogen mustards do not exclusively react with guanine.

More recently, a set of bis-3-chloropiperidines were reported based on the natural product 593A,^93^ a naturally occurring nitrogen mustard isolated from *Streptomyces griseoluteus*.^94^ Bis-3-chloropiperidines, including **B-CeP 1** (Figure 5c), showed efficient alkylation of a model DNA strand and cross-links were observed with only modest concentrations of 50 μM. Inspired by this, Sosic and co-workers applied **B-CeP 1** to investigate RNA tertiary structures.^95^ When tested on a model RNA construct, **B-CeP 1** rapidly alkylated RNA and substantial alkylation was observed after only 1 h incubation at 50 μM. Interestingly, no single strand breaks were observed in RNA, whereas a similar experiment with DNA yielded extensive backbone cleavage.^93^ To study RNA–RNA interactions, **B-CeP 1** was applied to the HIV-1 dimerization initiation site. Several inter- and intramolecular cross-links were detected using MS. Experiments were performed at both 25 and 95 °C to discriminate between these two types of cross-links. The apparent ability of **B-CeP 1** to cross-link duplex regions renders it an interesting tool to elucidate higher order structures.^95^

Modified nitrogen mustards have been developed to increase the functionality of these cross-linkers. In a recent study,^96^ a nitrogen mustard derivative was prepared bearing a cyclic disulfide group (**L1**, Figure 5c) that can be immobilized on a gold surface and used in Surface Plasmon Resonance (SPR). **L1** cross-linked a model DNA strand with ∼100% efficiency and was then used to measure cytosine methylation (mC) with SPR. Anti mC antibodies do not recognize 5-mC in duplex DNA, but do bind to 5-mC located in bulged dsDNA and the *K*_D_ was determined to be 7.70 × 10^–3^ using SPR. Interestingly, **L1** cross-linked and immobilized DNA showed a similar *K*_D_ of 5.60 × 10^–3^ toward anti-mC antibody enabling the analysis of 5-mC in genomic DNA. In a later study,^97^ the authors reported the design and synthesis of a biotin bearing nitrogen mustard **L2** (Figure 5c). The biotin provided enhanced functionality and cross-linked DNA was captured to streptavidin coated microtiter plates. Methylated cytosine was now quantified with an anti mC antibody and secondary antibody labeled with horseradish peroxidase. Using biological samples, the amount of 5-mC in mouse brain and intestine was determined to be 0.65% and 0.68% respectively. Although these examples were applied to DNA, we believe that they could find use in analysis of RNA structure as well.

### 2.5 Bifunctional Acylators

Early work by Knorre and co-workers^98^ showed that the 2′-OH of RNA is readily acylated when reacted with acetic anhydride in water at 0.25 M. Taking advantage of this, Weeks and co-workers invented SHAPE to deduce RNA secondary structure.^16^ When reacted with *N*-methylisatoic anhydride (13 mM), acyl adducts were left on flexible positions of RNA that were shown to stall reverse transcriptase. Using primer extension, the exact position of these adducts could be determined, which helped to deduce RNA secondary structures.^16^ This pioneering method has become widespread in the field and several new versions of acylating SHAPE reagents have been reported in the past few years.^18,99^ Furthermore, continued interest in these chemistries have provided new insight into differences in RNA reactivity toward 2′-OH acylating reagents.^100^

**Figure 6:**
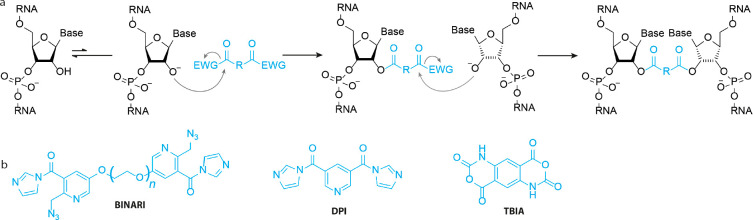
Bifunctional acylators. (a) Cross-linking mechanism. The 2′-OH attacks one of the carbonyls of the cross-linking reagent, while a second 2′-OH group that is close through space will attack the other carbonyl, effectuating a cross-link. EWG = electron withdrawing group (b) Molecular structure of bifunctional acylators.

Two consecutive acylating reactions on opposing nucleotides should result in a cross-link (Figure 6). Kool and co-workers explored this possibility using Bis-Nicotinic Azide Reversible Interaction (BINARI) probes (Figure 6b).^40^ The bifunctional probes bear two carbonylimidazoles that can react with the 2′-OH of opposing nucleotides. Depending on linker length between the reactive groups, cross-linking efficiencies between 45% and 84% were observed on a model self-complementary RNA duplex. To enable downstream analysis of cross-linked RNA, azide trigger groups were installed to reverse the cross-link. Azides could be reduced to amines using phosphines, promoting lactam formation and cross-link reversal. Reversal efficiencies ranged between 2% and 70% depending on phosphine, with 20 mM tris(hydroxypropyl) phosphine (THPP) being most efficient. The applicability of this cross-linking method was demonstrated by protecting RNA from nuclease digestion. A model RNA was fully degraded by S1 nuclease and RNase T1, whereas BINARI cross-linked RNA remained intact. Cross-link reversal by THPP liberated the model RNA strand, illustrating the potential use for chemical cross-linking for temporary RNA preservation.

In 2022, Lu, Velema, and colleagues explored the possibility of exploiting bifunctional acylators for RNA structure determination.^41^ This method, named Spatial 2′-Hydroxyl Acylation Reversible Cross-linking (SHARC), used simple dicarboxylic acids that could be activated with CDI in a single step to cross-link RNA. In particular, DPI (Figure 6b) efficiently cross-linked RNA up to 97%. To reverse the cross-link, mild alkaline conditions were used to hydrolyze the formed carboxylate ester, without affecting RNA integrity. Full cross-link reversal was observed in 100 mM borate buffer pH 10.0 for 2 h at 37 °C, with no apparent RNA degradation. Combining this with exonuclease trimming and proximity ligation, allowed for determination of RNA tertiary interactions (see section 3).

Weeks and co-workers developed *trans*-bis-isatoic anhydride^42^ (TBIA, Figure 6b) to cross-link 2′-OH of nucleotides that are close in space, which formed the basis for SHAPE-JuMP to interrogate RNA tertiary interactions. Applying TBIA to RNase P, ∼5–10% of RNA was cross-linked as apparent from lower mobility on PAGE. Using an engineered reverse transcriptase that can “jump” across the cross-link, the cross-linked sites were permanently recorded in the product cDNA strand, revealing tertiary RNA interactions.

Taken together, there are several options available when planning RNA cross-linking experiments. Historically, psoralens have been favored and continue to be indispensable tools for RNA structure determination. Advantages include relative selectivity for duplex regions and well characterized chemistry. Recent advances in 2′-OH acylators, provide an attractive alternative for psoralens. High cross-link and reversal efficiencies outperform most psoralen analogues. Current limitations are exclusive acylation of flexible RNA regions and monoacylation, complicating data analysis. We expect chemical improvements to address these drawbacks. Examples of aldehyde and nitrogen mustard cross-linking for RNA structure determination are scarce and the main drawback appears to be cross-link efficiency and reversibility for aldehydes and depurination and toxicity in the case of nitrogen mustards.

## 3 Measuring RNA Structures and Interactions with Chemical Cross-Linkers

**Figure 7:**
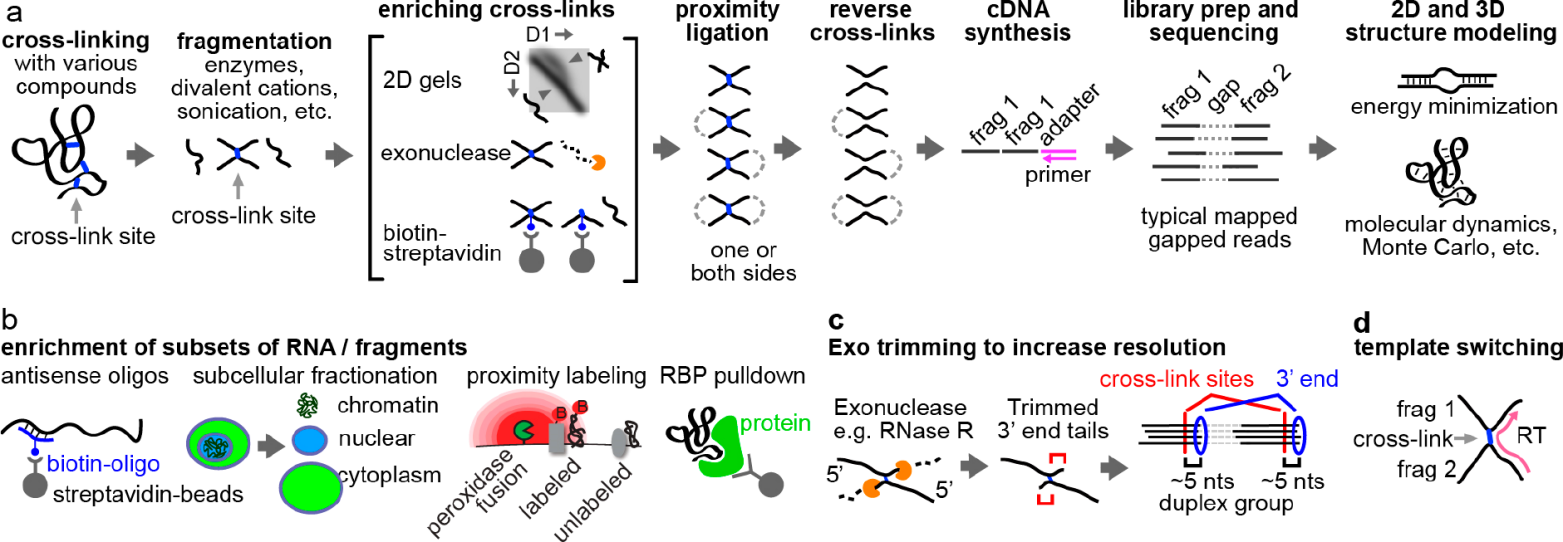
General workflow of cross-link-ligation methods. (a) Basic pipeline. RNAs are cross-linked, fragmented, and enriched for the cross-linked fragments using various methods such as 2D gels, exonucleases, or streptavidin-beads for biotinylated cross-linkers. After proximity ligation and reversal of cross-links, adapters are ligated to the fragments for cDNA synthesis, which is followed by cDNA amplification and high throughput sequencing. The gapped reads provide spatial constraints for 2D and 3D structure modeling. (b) Specific subsets of RNAs can be enriched for structure analysis after cross-linking and before fragmentation, using various approaches, e.g., antisense oligos, subcellular fractionation, proximity labeling (biotinylation by the APEX system) and antibody pull down of specific RNA binding proteins. (c) Exonuclease trimming can be used to improve resolution. Exonucleases are blocked by monoadducts or cross-links, leaving a stub of fixed length, and the cross-link sites can be deduced by counting backward from the 3′ ends. (d) Template switching is an alternative of proximity ligation to capture the two fragments in a single read, based on the ability of the reverse transcriptase (RT) to switch templates as it encounters roadblocks.

Once RNA has successfully been cross-linked, complexes can be analyzed using multiple different methods to identify the two cross-linked fragments. Classically, low-throughput methods have been used, including electron microscopy, gel electrophoresis, and low-throughput enzymatic sequencing.^101^ Development of high-throughput sequencing methods has enabled simultaneous measurement of transcriptome-wide RNA structures and interactions. Typical workflows of “cross-link-ligation-sequencing” methods include the following major steps: in vivo cross-linking, RNA fragmentation, enrichment of cross-linked fragments, proximity ligation, cross-link reversal, adapter ligation, reverse transcription, PCR amplification, and sequencing (Figure 7a). The gapped reads obtained after sequencing reveal base paired or spatial proximal RNA fragments, which are incorporated into 2D or 3D structure modeling, using various published computational tools.^22−24^ In addition to varying the choice of chemical cross-linkers described above, many variations of this general strategy have been reported. Here, we discuss these different options to enhance the workflow and focus on the enrichment of RNA types (Figure 7b), fragmentation by enzymes or ions, enrichment of cross-linked fragments (Figure 7a), approaches to increase resolution (Figure 7c), and alternatives to proximity ligation such as template switching (Figure 7d). These variations create a versatile toolbox, where different options can be selected for specific biological applications. Some of the most critical steps and options are discussed as follows.

### 3.1 Enrichment of RNA Subsets and Conformations

Abundance of cellular RNAs spans at least 7 orders of magnitude, making it difficult to study low abundance RNAs. In addition, RNA structure conformations can change during the life cycle of RNA biogenesis and function. To ensure sufficient RNA input, enriching subsets of the transcriptome is necessary. Several different approaches can be employed, including rRNA depletion (e.g., SPLASH, LIGR-seq),^64,71^ biotinylated antisense oligos targeting specific transcripts (e.g., PARIS, COMRADES),^60,66^ or antibody-based immunoprecipitation of protein-bound RNA (e.g., CLASH and hiCLIP) (Figure 7b).^102,103^ Subcellular fractionation using gradient centrifugation or the recently developed APEX proximity labeling offers another approach to enrich RNAs through different stages of their biogenesis or different subcellular localization.^104^

Each approach has its own advantages and problems. rRNA depletion or oligo(dT) enrichment of mRNAs may not be sufficient to isolate low-abundance RNAs. Biotinylated antisense oligos are expensive, making it hard to scale up the experiments. Antibody enrichment of ribonucleoprotein (RNP) complexes results in highly specific RNA conformations but depends on prior knowledge of the complex composition and the availability of high-quality antibodies. Centrifugation-based fractionation is very crude and highly variable, and often does not achieve sufficient specificity and purity. APEX-based enrichment introduces another irreversible chemical modification step that impedes reverse transcription (see further discussion below), reducing the efficiency of detecting proximally ligated RNA fragments.

### 3.2 Enrichment of Cross-Linked Fragments

Given the low efficiency of most cross-linking agents, many RNA fragments do not carry structural information and will ideally be removed. Several methods have been developed to enrich cross-linked fragments only, including 2-dimensional electrophoresis (2D gels), exonuclease digestion, and streptavidin selection of biotinylated cross-linkers. The 2D gel method, including both native-denatured 2D and denatured-denatured 2D, separates cross-linked fragments from non-cross-linked based on slower migration of its extended “X”-shape, which in theory provides 100% purity (Figure 7a).^60^ The 2D gel method is applicable to any chemical cross-linker, such as psoralens, nitrogen mustards and bifunctional acylators, since the separation only depends on RNA geometry.^60^ However, 2D gels are very laborious and difficult to scale up. Exonuclease digestion of non-cross-linked fragments is easier to perform, but also enriches non-cross-linked RNAs that are highly structured, have chemical monoadducts, or are cross-linked to proteins.^64^ Biotinylated cross-linkers allow facile enrichment of reacted RNAs using the biotin–streptavidin system (see examples in psoralens and nitrogen mustards in Figures 3 and 5). However, it not only enriches cross-linked fragments, but also monoadducts, which are likely more abundant than the cross-linked ones, leading to high background.

### 3.3 Exonuclease Trimming to Improve Resolution

Fragmented RNAs range in length from a few to several hundred nucleotides. While selection of shorter fragments increases the resolution of structure modeling, it only helps duplex modeling, where base pairing rules can be used to build the secondary structure model. Cross-linking of fragments that form tertiary contacts or spatial proximity does not provide sufficient resolution for structure modeling. To resolve this issue, two approaches have been developed. Exonucleases can trim off nucleotides from either end (e.g., RNase R for the 3′ end), until blocked by the cross-link, leaving a tail of a defined length (Figure 7c). Such tails can be used to determine the cross-linking sites precisely. However, monoadducts may also block the exonuclease, leading to incomplete trimming, and reducing precision. As an alternative to proximity ligation, sequence information from the two fragments can also be joined by template switching during reverse transcription (RT), especially with engineered reverse transcriptases that can jump across cross-linking junctions at higher frequencies (Figure 7d).^42^ This approach also improves the resolution in defining the cross-linked sites. However, this method has only been tested in simple RNA samples in vitro, and the efficiency is even lower than proximity ligation. Given that monoadducts affect both exonuclease trimming and template switching, further development of cross-linkers that produce minimal monoadducts is needed to improve resolution in the identification of cross-linking sites.

### 3.4 Proximity Ligation

Proximity ligation is the most widely used approach to join two cross-linked fragments. The ligation reaction can occur on either end, leading to different types of sequenced reads. Ligation of both ends leads to circular products that can no longer be ligated to adapters and are therefore lost during library preparation. However, given the low ligation efficiency, such double ligation events are rare and can be mostly ignored. The covalent linkage between the two fragments dramatically increases the likelihood of ligating them, compared to non-cross-linked ones in solution; however, the cross-linked stable structures and shortness of the fragments create steric hindrance, limiting ligation efficiency to typically below 15%. While longer fragments may be subject to lower steric hindrance and more efficiently ligate, they reduce the resolution of structure analysis. Several different protocols have been used in the last 10 years, including direct enzymatic ligation by T4 RNA Rnl1, Rnl2, CircLigase, Mth Rnl, and RtcB and the indirect ligation by incorporation of linkers, such as pCp-biotin and short oligos.^60,64,102,105^ Given that each protocol has several other steps that affect the perceived ligation efficiency, such as the purity of the cross-linked fragments (see the section above on enrichment of cross-linked fragments) and RNA damage levels, none of the improvements have been convincingly demonstrated to outperform others in side-by-side comparisons. New chemical and enzymatic approaches that overcome damages of RNA and steric hindrance of short fragments are needed to improve the ligation efficiency.

### 3.5 Reverse Transcription (RT)

**Figure 8:**
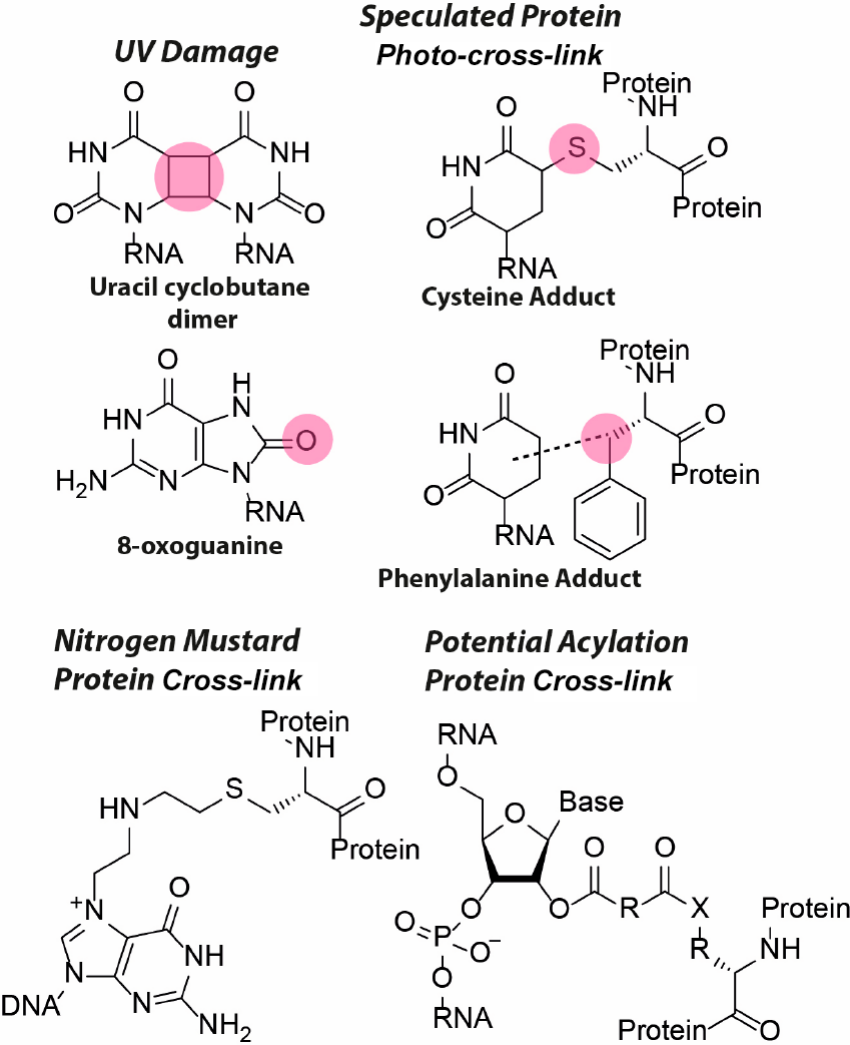
Molecular structure of side-products that can potentially interfere with reverse transcription. Multiple UV damage lesions have been reported with the uracil cyclobutane dimer and 8-oxoguanine as the predominant ones. Pyrimidines can photo-cross-link to proteins, with cysteine and phenylalanine adducts as speculated products. Nitrogen mustards can cross-link nucleic acids to cysteine residues. It is expected that bifunctional acylators can cross-link RNA to nucleophilic residues (X) on proteins.

Converting RNA to cDNA fragments is straightforward, but is impacted by side reactions of cross-linking, which reduce efficiency and accuracy of detecting the cross-linking events. Although more resistant to UV damage than DNA,^106^ UV-induced lesions in RNA are commonly observed and include base oxidation (primarily 8-oxoguanine),^107^ pyrimidine dimers,^107,108^ and adducts with proteins^109,110^ and other cellular components (Figure 8). Nitrogen mustards are highly reactive alkylating agents that can cross-link nucleic acids to proteins (Figure 8).^111^ While not reported yet, it is likely that bifunctional acylators can cross-link RNA to nucleophilic protein residues as well (Figure 8). Both lesions can potentially interfere with reverse transcription.

Several cross-linkers, such as nitrogen mustards and formaldehyde, suffer from poor reversibility, reducing the efficiency of cDNA synthesis and decreasing the perceived percentage of gapped reads if the RT is blocked before the ligation junctions. Damaged sites from 254 nm UV, psoralen and formaldehyde also lead to mutations and short 1–2 nt deletions, which are artifacts that confound the analysis of gapped reads.^60^ Improved RT conditions, such as the use of Mn^2+^ ions, and extended RT time, have been reported, but do not address all the different types of damages.^60,112^ In addition to developing better chemical cross-linkers and conditions to minimize damages, direct RNA sequencing may also help overcome some of these problems.^113^

## 4 Analysis of Chemical Cross-Linking Data and Structure Modeling

Despite the seemingly straightforward gapped reads from cross-link-ligation experiments, analysis of such data can be challenging. Numerous computational approaches have been described in the past few years. Here, we describe a unified conceptual framework and some of the critical considerations in the data analysis for various types of chemical cross-linking methods, focusing on two major steps: (1) extraction of information from reads including classification and clustering, and (2) inference of structural conformations, including 2D and 3D structure modeling. The first step processes the data, without the need for any prior knowledge or assumptions about the structure, whereas the second step models the structures based on principles of RNA folding.

### 4.1 Classification Read Types from Cross-Link-Ligation Experiments

**Figure 9:**
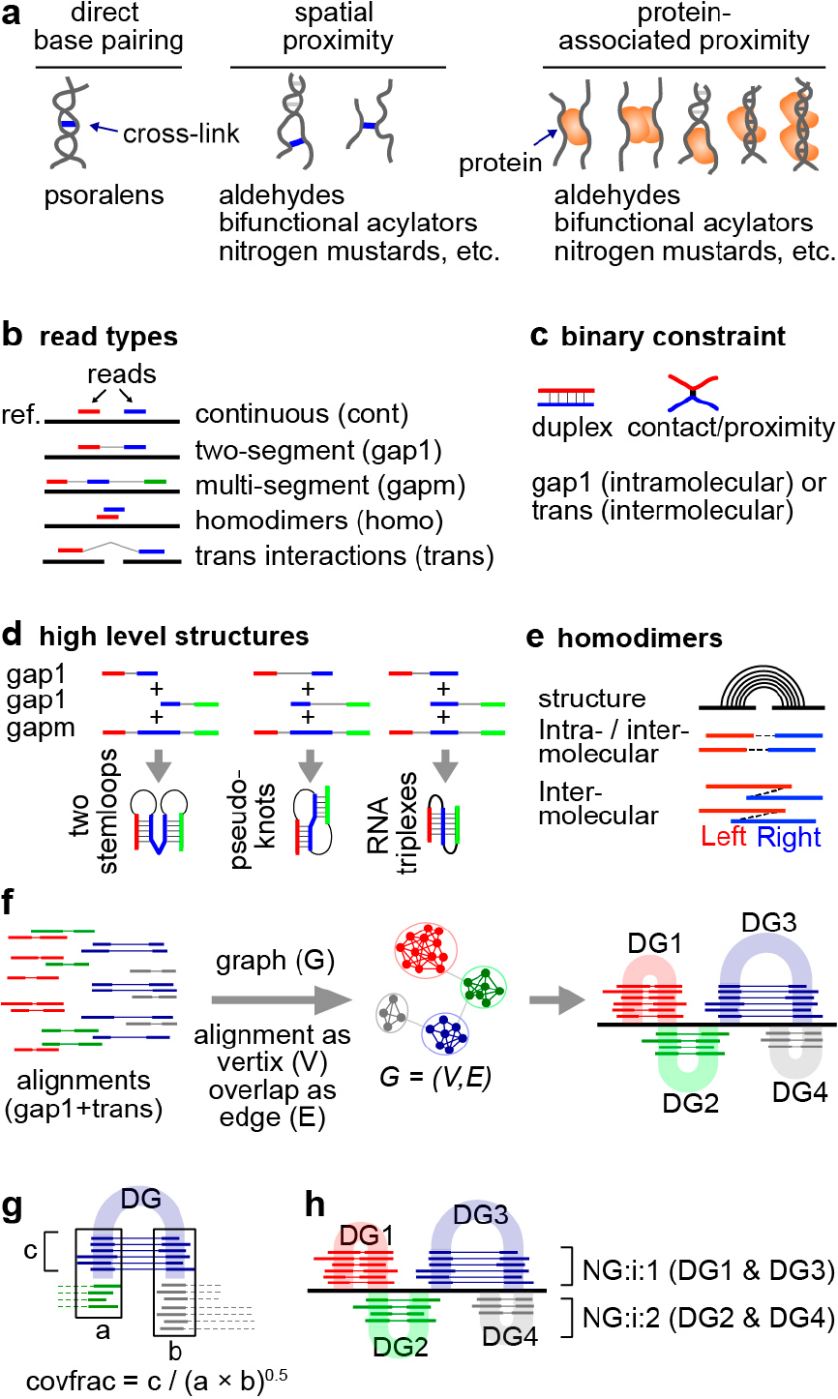
Strategies for extracting RNA structures and interactions from cross-link-ligation data. (a) Various types of cross-linked conformations by different chemical cross-linkers. (b) Classification and rearrangement of reads to 5 major categories, mapped to a reference genome (ref.). (c) Two basic types of binary constraints: basepaired duplex or contact/proximity. (d) Detection of high level structures by combining gap1 and gapm reads. (e) Detection of homodimers using reads with overlapped arms. (f) Clustering of gapped reads into duplex groups (DGs) using graph theory (network techniques). (g) Calculation of relative structure strength using the coverage fraction (covfrac) method. (h) Clustering and visualization of DGs into nonoverlapping groups (NGs).

In theory, cross-linking and ligation can occur on any RNA in physical proximity (as long as the chemistry of the cross-linker permits). Therefore, such experiments can join many different types of arrangements of RNA fragments into a single read. The chemical reactivities of the cross-linkers determine the types of structural information obtained (Figure 9a). For example, psoralens can only cross-link base paired regions, while aldehydes, nitrogen mustards and bifunctional acylators can cross-link any spatially proximal nucleotides, either constrained by nearby helices, tertiary contacts, or proteins. In addition, the ability of many RNA cross-linkers to react with proteins further increases the chances of capturing protein-mediated structures (Figure 9a). Psoralens can react with proteins, even though at much lower efficiency than nucleic acids, and lower compared to other cross-linkers.^114^ Proteinase treatment after cross-linking can exclude the protein-mediated proximities.^41,60^ Careful comparison of data from different types of cross-linkers will provide deeper insights into the organization of the RNP complexes.

Sequencing reads from cross-link experiments can be aligned to the reference using various types of mappers, such as STAR and Bowtie.^115^ Classification of these reads reveals the fragment arrangements and therefore the underlying structural conformations. Recent exhaustive classification results in 5 major read types (Figure 9b): continuous or nongapped reads that are due to failed cross-linking and/or ligation (cont), two-segment or single-gapped reads due to single ligation events either within one RNA (gap1) or between two RNA molecules (trans), multisegment (>2 segments) or multigapped (>1 gap) reads due to simultaneous multi-cross-link and multiligation events on several RNA fragments in spatial proximity (gapm), and overlapping fragments that come from RNA homodimers (homo).^60,116,117^

Reads with a single gap (gap1 and trans) suggest a single constraint in the structure, either a double helix cross-linked by psoralens, or tertiary contact/proximity cross-linked by SHARC or TBIA and other types of cross-linkers (Figure 9c). Reads with more than one gap suggest more complex structures, such as consecutive stemloops, pseudoknots and triplexes, depending on the relative location of the two stem regions (Figure 9d). Coassembly of the gap1 and gapm reads provide strong evidence for these more complex structures. The percentages of gapm reads are very low in current methods due to the low cross-linking and proximity ligation efficiencies, limiting the discovery of complex conformations. A small percentage of reads have two segments overlapping each other. This is not possible for an intramolecular duplex since the fragmentation should only remove parts of the RNA. The only explanation is that the two fragments came from two identical RNA molecules, in other words, RNA homodimers (Figure 9e).^116^ Additional types that are combinations of these 5 types are also possible and indicate even more complex structural conformations.

### 4.2 Clustering of Reads into Groups That Represent Specific Structures

High-throughput sequencing produces very dense gapped reads that come from various structural units and conformations in the transcriptome, making it difficult to interpret the data. Based on the assumption that each structure unit produces a set of reads that are highly similar to each other, the reads can be clustered, where each group can be used to infer a specific contact (or closely positioned contacts, Figure 9f). This approach was first proposed in 2016 and has been systematically benchmarked, optimized and widely applied to various experimental methods.^33,41,63,105,116^ The clustering of multiple different DGs provides direct evidence for the existence of alternative or dynamic conformations. Such conformations are easily detected for psoralen cross-linked RNA fragments, given the mutually exclusive base pairing (except the triplexes and G-quadruplexes where each stretch of nucleotides can participate in multiple consecutive contacts). Other clustering approaches have been developed simply based on overlap of arms among reads.^118^

The clustering approach provides a statistical assessment of the underlying structure (Figure 9g) and a method for visualization (Figure 9h). RNA structural conformations exist in variable frequencies, and are cross-linked at different rates, resulting in read groups with a wide range of abundances. To quantify the abundance, coverage fraction can be used, where the read number in each group is normalized by the total coverage of reads across the two arms (Figure 9g). Several different types of statistical tests of the significance for the structure formation can be used to rank and filter data, such as binomial and Fisher’s exact test, which typically assess the significance of the ligation event against expression levels of the RNAs.^118,119^ For efficient visualization, the DGs are further arranged into nonoverlapping groups (NGs), enabling tight packing of the reads in genome browsers (e.g., IGV,^120^Figure 9h). This visualization of the read groups does not make assumptions about the underlying experimental methods and does not require knowledge-based inference of structures, and thus is generally applicable to all types of cross-linking data and free from modeling biases. The initial implementation considers only RNA double helices based on gap1 reads, hence the name duplex group (DG). However, the basic principle has been extended to include multigapped reads (gapm), homodimers (homo), which are technically the same as typical heteroduplexes, and binary nonbasepairing spatial proximity or contacts (e.g., from other cross-linkers beyond psoralens).

### 4.3 Structure Modeling Assisted by Experimental Constraints

**Figure 10:**
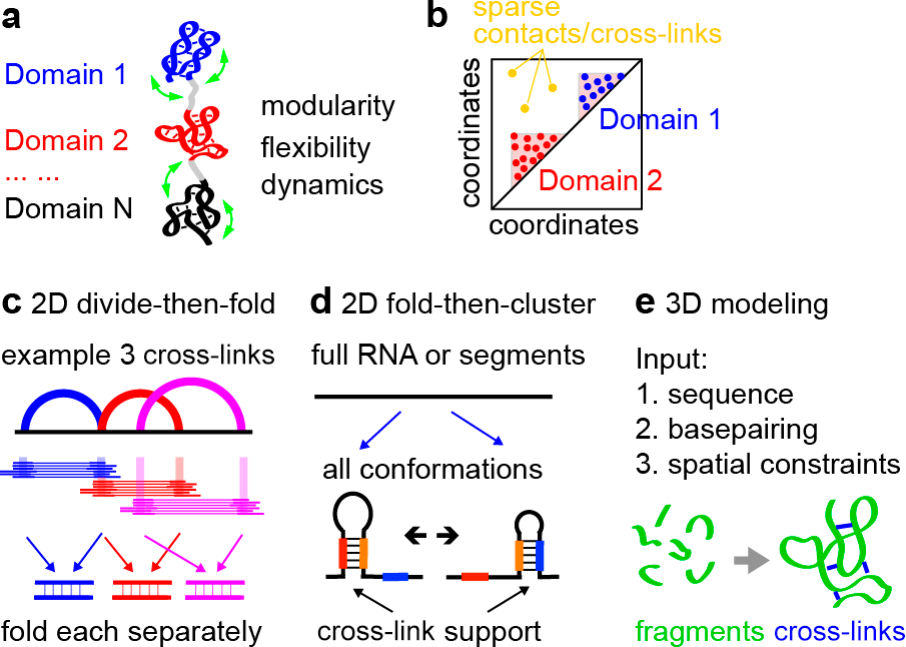
Modeling of structures from cross-linking constraints. (a) Conceptual diagram of high-level modular RNA domains that are flexible and dynamic. (b) Clustering of contacts define RNA domains. (c) Divide and then fold approach for secondary structure modeling, exemplified by 3 cross-links. (d) Fold and then cluster approach for secondary structure modeling, for 2 example alternative conformations. (e) Typical fragment assembly-based approach for 3D modeling, where known 3D fragments are assembled and constrained by cross-linking data (e.g., Rosetta).

Protein-mediated proximities and in situ proximity ligations can join any RNA fragments as long as they are close to each other in space, but does not require direct contact, making it difficult to determine the RNA conformations (Figure 9a). Direct RNA cross-linking, such as by psoralen and bifunctional acylation reagents (e.g., SHARC and TBIA), especially after proteinase treatment to remove proteins, captures direct secondary and tertiary contacts/proximity which are more useful for structure modeling (Figure 9a). At a higher level, many recent studies have revealed modular domains in RNA architectures, defined by frequent cross-links within each domain and sparse cross-links between domains (Figure 10a,b).^63,66,121−123^ The large domains are linked by more flexible and dynamic regions. The discovery of these modular domains based on many different types of chemical cross-linkers (e.g., psoralen and formaldehyde) demonstrated their authenticity. At a lower level, the inference of individual secondary and tertiary structure units from cross-linking data remains an unsolved problem, for several reasons. First, the low cross-link-ligation efficiency and strong bias led to incomplete data for modeling (e.g., psoralen bias toward uridines, and acylation bias toward unconstrained riboses). Second, conventional computational modeling tools were not optimized for the incorporation of cross-link-ligation data, so it remains unclear how to make best use of experimental constraints. Third, current modeling tools can only be applied to short RNAs due to the prohibitive computational cost. Despite these challenges, several general approaches have been implemented.

#### 4.3.1 2D Structures

Two different strategies are used for 2D structure modeling based on experimental constraints: divide-then-fold (Figure 10c), and fold-then-cluster (Figure 10d). In the divide-then-fold approach, the gapped reads (and the assembled DGs) carve out pairs of RNA fragments, and minimal free energy structures can be built from the two fragments (Figure 10c).^63^ Whole transcript structures can then be established by combining all the structure models. This method is fast, since only cross-linked regions are modeled, however, the resulting global conformations cannot be clearly deconvolved. Typically, one structure map is produced for each RNA as a result, including all the predicted helices, some of which may be mutually exclusive (Figure 10c, blue and red arcs with overlapping arms). Alternatively, in the fold-then-cluster approach, RNA molecules can be folded de novo, producing an ensemble of global conformations, which are then clustered (Figure 10d).^66,124^ The gapped reads (and the assembled DGs) are then mapped onto the clustered conformations. This approach outputs complete global conformations, however, it also suffers from several caveats. The structures may be overfolded, and not all duplexes in such models are necessarily supported by experimental constraints. The folding step may not be feasible for long cellular and viral RNAs, many of which measure tens to hundreds of thousands of nucleotides. As a result, folding is often performed in windows of limited lengths (e.g., 300–1000 nts), which excludes long-range structures. In the future, integration of these two approaches is needed to increase the efficiency and accuracy of 2D structure modeling. To quickly validate and rank the biological relevance of these secondary structure models, multiple sequence alignments and identification of conserved and covaried base pairs are often performed, and compared to experimental constraints.^63,122^

#### 4.3.2 3D Structures

Computational modeling of 3D RNA structures is a rapidly progressing field, and many different approaches have been proposed, such as molecular dynamics, fragment assembly, and deep learning (Figure 10e).^14^ In addition to the primary sequence and secondary structure models, experimental constraints are typically incorporated into the modeling as pseudoenergy terms: predicted conformations are penalized where the constraint is not satisfied. Compared to 2D modeling, the basic rules in 3D structure modeling have not been thoroughly studied,^125^ and therefore, the modeling results are typically confounded by multiple sources of error, both computational and experimental. Despite that experimental constraints can be obtained for RNAs of any length, modeling of large RNAs remain impractical due to the high computational cost.

## 5 Biological Applications: Critical Considerations and Recent Examples

Cross-link-ligation methods have a wide range of applications in RNA biology and medicine. The earliest applications of these chemical tools were critical in establishing the structural mechanisms of pre-mRNA splicing and rRNA processing. Here we discuss critical considerations in applying these methods, and review some of the most interesting examples in recent years, focusing on lncRNAs, ncRNA networks, genetic disorders and RNA viruses, which will help researchers to properly apply them to their biological problems.

### 5.1 Structure-Guided Studies of RNA Functions: Choosing Proper Methods and Important Biological Targets

Recent development of sequencing-based strategies allowed deeper interrogation of transcriptome-wide RNA folding principles, lower abundance RNAs and low frequency alternative/dynamics conformations. While general properties of RNA folding can be extracted from transcriptome-wide measurements, rigorous functional studies of the newly discovered RNA structures and interactions have not caught up with the rapidly growing large lists of new data from high-throughput sequencing. The choice of chemical cross-linkers should be based on the needs of specific biological questions. For example, detection of base pairing mediated structures and interactions require psoralens, whereas general spatial proximity can be studied using other cross-linkers (Figure 9a). Even for chemical cross-linkers that detect general spatial proximity, proteinase K treatment is necessary to exclude protein-mediated conformations (Figure 9a). Multiple different types of cross-linkers may be needed to obtain more details regarding complex RNP complexes, such as 2D structures, 3D structures and RNA-protein interactions.^126,127^

To pursue discovery-driven studies of RNA structures, several important aspects need to be considered even if the sequencing experiment was designed to test a specific hypothesis as opposed to an unbiased discovery. First, analysis of the relative coverage and statistical significance is needed to predict the biological significance of the newly discovered structures or interactions, as described above (Figure 9g).^118,119^ More abundant and reproducible structures are more likely to contribute to functions. Second, validation by various other methods, especially structure conservation/covariation increases the confidence in the functional significance.^128^ Third, direct connection of the structural elements to functional sequence motifs (e.g., in splicing regulation), protein binding sites, RNA modification sites and processing patterns, etc. indicates potential functions in these aspects. Last, linkage of the structures to human diseases is a strong motivation for further mechanistic analysis. These principles have been employed in several recent studies. After discovery of potentially important RNA structures, care should be taken in studies of their function, since functions for one specific DNA and its RNA products can be encoded on multiple levels, such as DNA sequences, RNA sequences, RNA–protein or RNA–RNA interactions, as well as RNA structures. To demonstrate that functional consequences are indeed due to structure formation, multiple types of mutation and compensatory rescue tests are needed.

### 5.2 Solving In Vivo RNA Structures and Interactions to Understand RNA Functions: Recent Examples

Recent applications of the cross-link-ligation methods have been focused on several directions, including RNA virus genome structures and host–virus interactions,^66,129−131^ noncoding RNA interactions^116^ and lncRNA functions.^63,121,132^

Single-stranded RNA viruses are a major target of the cross-link-ligation methods, because of the strong dependence of the viruses on their genome structures and RNA-mediated host–virus interactions, and the therapeutic potential of targeting viral RNA genomes. Recent studies have reported genome structures and host–virus interactions for *Flaviviruses*, such as Zika and Dengue,^66,122^*Coronaviruses*, such as SARS-CoV-2,^129,131^ and *Picornaviruses*, such as EV-D68.^60^ These studies confirmed many previously predicted structures, revealed new structural elements and new miRNA and snoRNA-mediated host–virus interactions that affect virus fitness.

The transcriptomes of most organisms contain a large number of short noncoding RNAs that regulate other RNAs through base pairing. Applications of the cross-link-ligation methods have led to the discovery of interaction networks for a variety of species and include bacterial sRNAs,^133^ eukaryotic miRNAs^134^ and snoRNAs.^60,66,116^ These studies dramatically expanded the known targets of important ncRNA regulators. Given the low abundance of many ncRNAs and their targets, it is expected that future deeper sequencing, coupled with RNA enrichment methods (Figure 7), will continue to reveal new targets. Application of these methods to other recently characterized ncRNAs, such as tRNA fragments and rRNA fragments, will help decipher their functions, most of which remain unknown up to now.

LncRNAs are a diverse class of regulatory RNAs with broad roles in gene expression control and other cellular processes. Cross-link-ligation methods have led to new insights into their mechanisms of action, including the discovery of modular domains that organize their multiple functions and spatial separation of these functions.^63,121,132,135^ Such studies provide a proof-of-principle for future analysis of other large RNAs, including mRNA UTRs and introns, whose primary functions are noncoding and thus may use similar mechanisms.

## 6 Conclusions and Perspectives

In the past 10–20 years, extensive technological improvements of 1D chemical probing methods have led to their widespread applications in various biological systems. Despite the rapid progress in the field of RNA cross-linking in recent years, there remain several technical challenges in the study of RNA structures and interactions in living cells. Cross-linking-based methods are difficult to implement due to the inefficiencies at multiple steps and complexities of computational analysis, which creates a major barrier for its wide adoption. Given that most of these methods were only recently developed, evaluation of the computational tools has not been performed. More efficient tools will likely be needed to tackle challenging biological problems including RNA structural dynamics and heterogeneity in space and time, such as embryonic development and disease, large RNAs and their complexes with other RNAs and proteins, and high resolution structure modeling. At the same time, systematic side-by-side comparison of different methods will help clarify their strengths and weaknesses, increase the efficiency of these methods and make it possible for more laboratories to implement them.^60^ Here, we provide some perspectives on further technology development in this field.

### 6.1 New RNA Cross-Linking Chemistry: Faster, Less Bias, More Precise, and More Efficient

Even though a wide variety of physical and chemical cross-linkers are available now, there are still several unsolved problems, including the cross-linking efficiency, side reactions, bias, and reaction kinetics. In vitro tests have shown that SHARC reagents are the most efficient, but they are not reactive toward tightly packed structures in cells.^41^ In other words, they are biased toward single stranded and unconstrained nucleotides. All current cross-linkers, except direct UV, are relatively slow and typically require at least 10 min to achieve sufficient efficiency for subsequent sequencing experiments. Structural dynamics in cellular RNAs can span 18 orders of magnitude, down to picoseconds.^136^ While the ultrafast dynamics can only be studied using physical methods in vitro, some of the structural transition dynamics that range from seconds to minutes are potentially tractable using faster chemical cross-linkers. Several new chemical tools have been reported recently to improve the temporal resolution in 1D chemical probing and one-sided cross-linking, such as nicotinoyl azide and cyanovinyl carbazoles, offering interesting new ideas for developing new RNA cross-linkers.^137^ Achieving both fast kinetics and reversibility in the same bifunctional cross-linker is still challenging.

Another important shortcoming that potentially can be addressed with improved chemistry is the existence of monoadducts that complicate analysis. Recent advances in the field of self-immolative linkers^138,139^ may provide inspiration for chemical cross-linkers that display significantly fewer monoadducts.

### 6.2 Long-Read Sequencing to Capture Whole-Transcript Structures

Current ligation and template switching based methods can only capture small structure units, such as duplexes, even though they are not constrained by sequence length. Simultaneous cross-linking and ligation have enabled the discovery of combinations of the duplexes, including consecutive stemloops, pseudoknots and triplexes (Figure 9d).^116^ Given the large number of possible conformations for each RNA species, it is currently impossible to stitch together the structural units into complete models for whole transcripts. Long-read sequencing has been used to capture potentially full-length or longer conformations from foot-printing based chemical probing methods.^127^ Dramatically improved cross-linking and ligation efficiency may produce longer RNA fragment combinations, which together with long-read sequencing, can link co-occurring and distinguish mutually exclusive conformations.

### 6.3 Single-Cell Analysis of RNA Structure Heterogeneity

Alternative RNA structures are pervasive in cells, and likely contribute to cell-type-specific regulation of gene expression. Indeed, such examples have been demonstrated in RNA-structure-dependent alternative splicing of cell adhesion molecules that determine neuronal connectivity, such as insect *Dscam* and mammalian *Neurexins*.^140^ Even though splicing-regulatory intronic structures have been discovered, little is known about their cell type specificity and whether they correlate with the splicing outcome in individual cells. Development of single cell structure mapping methods will be necessary to link the structural conformations to the functional outcomes. Despite the success of single cell RNA-seq, structure analysis is more challenging. First, the low sequencing coverage in individual cells make it hard to get sufficient reads to build structure models. Targeted enrichment of RNA species will be necessary to obtain sufficient data. Second, incorporation of the cross-link-ligation protocol into single cell sequencing workflows is not trivial. Some of the critical steps in the cross-link-ligation methods, such as enrichment of cross-linked fragments (2D gels and biotinylated cross-linkers) have been only implemented in bulk samples.

### 6.4 Computational Integration of Various Structure Mapping Methods

The cross-link-ligation data provide constraints for global organization of RNA structures but lack sufficient resolution on the details. Even though the exo trimming and template-switching methods can pinpoint some of the cross-linking sites (PARIS-exo, SHARC-exo and SHAPE-JuMP),^41,42^ not every base pair can be identified with high confidence. On the other hand, 1D chemical probing data, such as from SHAPE and DMS-seq, provides detailed information on the structure/interaction constraints on individual nucleotides, despite the lack of confidence in their discovery of complex and long-range structures. New computational tools that carefully evaluates data from various experimental methods are needed to effectively take advantage of each method and avoid artifacts. With new computational modeling algorithms, 1D accessibility, 2D base pairing and 3D spatial proximity constraints should enable better modeling of RNA structures.

### 6.5 High-Resolution and High-Level Structure Modeling

The long-term goal of developing cross-link-ligation and other experimental tools is to build high resolution and comprehensive structure models for RNA, which will hopefully provide new insights into their functions in vivo. While many computational tools have been developed to build 2D and 3D structures, such tools are often developed based on energy terms and parameters derived from a small number of in vitro models. Furthermore, RNA structural dynamics is not only determined by its primary sequence, but also cellular environments, such as proteins, other RNAs, ions, metabolites, temperature, and pH, most of which cannot be incorporated into the modeling studies.

Earlier studies have made use of in vitro constraints from correlated chemical probing to improve computational modeling.^141^ Now, the cross-linking-derived in vivo constraints offer a unique opportunity to model 2D/3D RNA that represent true in vivo conformations. On the experimental side, distance measurements are not always accurate. Exo trimming is effective but is can be problematic due to monoadducts blocking the exonuclease. The template switching strategy in SHAPE-Jump is another approach but suffers from high noise, due to the low efficiency and randomness of template switching.^42^ Even though chemical cross-linking indicates a probable distance between two nucleotides, the range of the distance distribution can be quite large due to the natural dynamics of the RNA in cells. Furthermore, very few RNA molecules exist in their naked form; most RNAs are bound by a wide variety of proteins. Ignoring the proteins in the modeling is bound to introduce errors. On the computational side, current implementation of such approaches are still rudimentary, and only produce very simple models.^41,42^ New methods that consider the in vivo flexibility and dynamics during modeling are needed to produce ensembles of structure conformations that are more biologically relevant. Conventional 3D modeling tools are extremely computationally expensive, currently only able to handle RNAs within 200–300 nucleotides. Faster new algorithms are needed for the vast majority of the large RNA molecules in cells, which often span hundreds to tens of thousands of nucleotides.

### 6.6 Discovery of In Vivo RNA Structures and Interactions for Targeted Therapeutics

Normal and abnormal functions of RNA molecules have been implicated in many human diseases, such as nucleotide repeat disorders, RNA virus infections, and splicing mutations, which account for at least a third of all disease mutations. The past decade has witnessed a revolution in the field of RNA therapeutics, with the approval of multiple small molecule and antisense oligo drugs that target a wide variety of RNA sequence and structural elements with critical roles in human diseases.^142^ Many more RNA-targeting therapeutics are either in preclinical studies or clinical trials. Structural studies have played critical roles in some of these RNA therapeutics and we expect that the increasing availability of RNA cross-linking experiments will further assist toward this goal in the future. For example, the discovery of intronic sequence and structure elements in the SMN2 gene led to the development of antisense oligo drugs to treat spinal muscular atrophy, one of the most prevalent pediatric genetic disorders.^143^

In summary, we hope that our critical review of chemical cross-linking methods, including the chemistry, enzymology, computational analysis, and biological applications, will spur further improvements of these methods and applications in chemistry, biology, and therapeutics. Improved structural information will bring conventional sequence motif-based studies of RNA regulation to higher dimensions in the future. Better structure models obtained with enhanced chemical cross-linkers will provide essential guidance for the development of RNA-based and RNA-targeting drugs.
